# Case Report: Chronic myeloid leukemia masquerading as essential thrombocythemia in a child: the imperative for early BCR::ABL1 testing in isolated thrombocytosis

**DOI:** 10.3389/fped.2026.1846571

**Published:** 2026-07-20

**Authors:** Haoping He, Jinqiu Fu

**Affiliations:** Department of Pediatrics, Qilu Hospital of Shandong University, Jinan, Shandong, China

**Keywords:** BCR::ABL1, case report, chronic myeloid leukemia, isolated thrombocytosis, pediatrics

## Abstract

Chronic myeloid leukemia (CML) is a rare hematologic malignancy in children, typically presenting with leukocytosis in the peripheral blood, and often accompanied by symptoms such as fever or splenomegaly. This report describes a rare pediatric case of CML that manifested solely as isolated thrombocytosis. Due to this atypical presentation, the patient was initially misdiagnosed with essential thrombocythemia (ET) and treated with hydroxyurea and aspirin, which yielded no clinical response. The diagnosis of CML was subsequently confirmed by detection of the BCR::ABL1 fusion gene. Upon confirmation, the patient was promptly initiated on imatinib therapy and achieved a complete cytogenetic response within 4 months. We also conducted a literature review of similar cases, discussing their prognosis and differential diagnosis with ET, and recommend early BCR::ABL1 fusion gene testing in all pediatric patients presenting with isolated thrombocytosis to ensure accurate diagnosis.

## Introduction

Chronic myeloid leukemia (CML) is a rare hematologic malignancy with an incidence rate of approximately 2 per 100,000 (1). Its incidence correlates positively with age, with pediatric CML occurring at a rate of about 0.6–1.2 per 1 million, accounting for only 2%–3% of all childhood leukemias (2). In China, the overall population incidence of CML is approximately 0.7–0.9 per 100,000 (3). The hallmark of CML is the presence of the Philadelphia chromosome or the BCR::ABL1 fusion gene. Clinically, pediatric CML lacks distinctive specificity; some children present with fever, fatigue, and splenomegaly, while others remain asymptomatic. Laboratory findings typically reveal marked elevations in white blood cells and granulocytes, occasionally accompanied by anemia and mild thrombocytosis (4, 5). Consequently, the onset of pediatric CML is often insidious. Diagnosis typically arises when abnormal blood counts or splenomegaly detected during routine physical examination raise suspicion of a hematologic disorder, prompting confirmatory testing-including bone marrow morphology, chromosomal karyotyping, and fusion gene analysis. However, in a very small number of cases, the condition may present solely as marked thrombocytosis without abnormalities in the peripheral white blood cell count, posing a significant diagnostic challenge. This report documents a rare pediatric case in which marked thrombocytosis was the sole initial clinical manifestation. A literature review explores diagnostic considerations for such atypical CML presentations, offering insights to aid clinicians in timely recognition and diagnosis.

## Case description

A 12-year-and-6-month-old female patient presented at a local hospital in June 2025 with complaints of “irregular menstruation and prolonged menstrual periods”. A complete blood count revealed anemia and a mild increase in platelet count. The anemia was attributed to prolonged menstrual bleeding. Following treatment with progesterone, menstruation ceased and anemia improved significantly. However, follow-up laboratory results showed persistent thrombocytosis (platelet count: 900–1,100 × 10^9^/L). (Table 1) For further evaluation, the patient was referred to our hospital in July 2025. During the consultation, we performed a detailed physical examination of the patient and found no abnormal findings, such as hepatomegaly, splenomegaly, or petechiae. We also inquired about the patient's recent medical history and family history. The patient reported no recent history of infection, inflammation, connective tissue disease, or surgery, and denied any family history of hereditary thrombocytosis or other myeloproliferative disorders. The peripheral blood smear results show occasional neutrophil myelocytes; the percentages and morphology of cells in each lineage are generally normal, and clumped platelets are frequently observed. Bone marrow morphology examination revealed: >1000 megakaryocytes per smear, frequent platelet clusters, elevated white blood cell count, and occasional metamyelocytes, with lineage ratios and cellular morphology generally within normal limits. Bone marrow biopsy showed active hyperplasia (approximately 90%) with trilineage proliferation myeloid, erythroid, and megakaryocytic with increased megakaryocytes. (Table 2) Concurrent genetic testing yielded the following results: whole-exome genetic testing did not identify any gene mutations with clear clinical significance. No mutations were detected in JAK2 exon 12, CALR exon 9, or MPL exon 10. Based on these findings, the patient was initially diagnosed with thrombocytosis and treated with oral aspirin and hydroxyurea for three months, with suboptimal response. During this period, platelet counts remained markedly elevated and highly variable (ranging from 648 to 2,377 × 10^9^/L). The inadequate response to conventional essential thrombocythemia (ET) therapy raised suspicion of an alternative diagnosis, prompting further investigation.

**Table 1:** Blood count results of the patient.

Date	WBC (×10^9^/L)	NEU (%)	EOS (%)	BAS (%)	Hb (g/L)	PLT (×10^9^/L)
June 08, 2025	3.64	59.70	1.30	0.15	85	611
June 17, 2025	4.99	63.40	0.44	0.00	96	901
June 29, 2025	8.48	55.60	1.20	0.20	113	1,096
July 12, 2025	6.53	61.70	1.40	1.30	106	1,062
July 30, 2025	14.35	69.90	1.90	0.70	125	1,466
August 26, 2025	2.30	42.50	1.30	3.50	113	648
September 14, 2025	3.74	51.80	2.10	0.10	122	1,848
October 03, 2025	3.95	60.50	1.15	0.75	128	2,377
October 20, 2025	4.50	54.10	3.00	6.80	118	1,165
October 29, 2025	8.81	64.40	0.70	0.50	119	483
November 12, 2025	2.81	64.30	2.00	0.90	113	55
December 06, 2025	3.15	59.60	1.40	0.70	125	241
January 10, 2026	5.03	54.90	1.60	0.20	121	165
January 17, 2026	8.12	62.80	1.20	0.00	118	127
January 24, 2026	10.91	28.10	0.37	0.05	132	286
January 30, 2026	4.70	54.30	0.10	0.00	108	208
February 28, 2026	4.67	47.60	2.20	0.20	109	165

WBC, white blood cell; NEU, neutrophil; EOS, eosinophil; BAS, basophil; Hb, haemoglobin; PLT, platelet.

**Table 2:** Bone marrow morphology and biopsy results at initial presentation.

Bone Marrow Morphology			
Cell Type			Percentage (%)
Myeloid	Neutrophils	Myelocyte	8.0
		Metamyelocyte	11.0
		Band neutrophil	12.0
		Segmented neutrophil	11.0
	Eosinophils	Myelocyte	<1.0
		Metamyelocyte	<1.0
		Band neutrophil	1.0
		Segmented neutrophil	1.0
	Basophils	Myelocyte	<1.0
		Metamyelocyte	<1.0
		Band neutrophil	<1.0
		Segmented neutrophil	<1.0
Erythroid		Pronormoblast	<1.0
		Basophilic erythroblast	1.0
		Polychromatophilic erythroblast	5.0
		Metarubricyte	6.0
Myeloblast			1.0
Microscopic morphology	Each smear contained more than 1,000 megakaryocytes; no morphological abnormalities were observed in the megakaryocytes; platelet clumping was common; the white blood cell count was elevated; medullary cells were occasionally observed; and the proportions and morphologies of all blood cell lineages were generally within the normal range.		
**Biopsy Results**			
Microscopic morphology	HE and PAS staining revealed that the submitted bone marrow sample exhibited relatively active proliferation (approximately 90%), with a slightly increased granulocyte-to-erythrocyte ratio. Cells at all stages of the granulocyte lineage were present, predominantly at the intermediate and early stages or below; eosinophils were readily visible. Cells at all stages of the erythrocyte lineage were present, predominantly intermediate and late erythroblasts. Megakaryocytes were increased in number, distributed sporadically or in clusters, with segmented nuclei; megakaryocytes with small cell bodies and few lobes are also present; a small number of lymphocytes and plasma cells are distributed sporadically or in small clusters, and are morphologically mature		
Myelofibrosis (MF) grade	MF-0		

The patient returned to our hospital for follow-up in October 2025, and a second bone marrow aspiration was performed. Morphological assessment again revealed active proliferation with abundant platelets. Given the lack of therapeutic response over the preceding three months and to exclude an occult hematologic malignancy, bone marrow immunophenotyping was conducted: CD34^+^CD117^+^ immature myeloid cells accounted for 1.15% of nucleated cells, indicating an elevated proportion; a subset of these cells aberrantly expressed CD7. Additionally, CD19^+^CD10st^+^CD34^part+^ immature B cells constituted 0.06% of the population, exhibiting enhanced CD45 and diminished CD38 expression-features consistent with phenotypic abnormality. Subsequent fusion gene testing confirmed BCR::ABL positivity. In the bone marrow sample, BCR::ABL/ABL (copy number) was 31.5% (international scale); in peripheral blood, it was 23.6% (international scale). Conventional karyotype analysis yielded a normal female karyotype: 46, XX. However, FISH analysis for the BCR::ABL1 fusion gene was positive. Based on these integrated findings, the patient was definitively diagnosed with CML and initiated on oral imatinib therapy on October 25, 2025, at an initial dose of 200 mg/day (adjusted for body weight of 40 kg). Follow-up blood tests demonstrated a gradual decline in platelet counts to normal levels (nadir: 55 × 10^9^/L). Imatinib dosage was dynamically adjusted based on serial platelet monitoring, with a final stable maintenance dose of 200 mg/day, sustaining platelet counts within the normal range of 100–200 × 10^9^/L.

In February 2026, the patient returned to our hospital for a follow-up examination. Fusion gene testing of bone marrow revealed BCR::ABL(P210) fusion mRNA by quantitative real-time RT-PCR. The BCR::ABL/ABL ratio was 0.27% (copy number, international standard), while peripheral blood showed a BCR::ABL/ABL ratio of 0.19% (copy number, international standard). Flow cytometry indicated that CD34^+^CD117^+^ myeloid blast cells constituted 0.42% of the bone marrow sample, and CD34^−^CD117^+^ myeloid immature cells accounted for 0.59% of nucleated cells. No abnormalities were observed in either proportion or immunophenotype. Bone marrow morphology demonstrated active hyperplasia with 10^6^ megakaryocytes per high-power field and scattered platelets, consistent with post-treatment CML.

The patient is currently receiving oral imatinib on a regular schedule. Blood tests show stable hematologic parameters and a marked reduction in BCR::ABL fusion transcript levels in both bone marrow and peripheral blood compared to prior measurements. Based on the above evaluation results, the patient has achieved complete cytogenetic remission (CCyR) after nearly 4 months of treatment, and the treatment response at this stage is assessed as optimal. The patient remains under ongoing outpatient management at our institution.

## Discussion

With tyrosine kinase inhibitors (TKIs) now established as first-line therapy, the overall prognosis for pediatric CML has improved substantially. Recent studies report that children treated with imatinib as initial therapy achieve a 36-month overall survival rate of 97.4% (6). In this context of favorable outcomes, therapeutic strategies for pediatric CML are increasingly focused on shortening TKI treatment duration and minimizing adverse effects, offering an encouraging clinical outlook (7, 8). However, a minority of CML cases lack distinctive clinical features, complicating early diagnosis. The case described herein initially presented without physical examination abnormalities, with isolated thrombocytosis as the sole hematologic finding. CML manifesting exclusively as isolated thrombocytosis is exceedingly rare and poses significant diagnostic challenges. Given the limited clinical experience with such presentations, clinicians often express concern regarding the potential consequences of delayed or incorrect diagnosis on treatment initiation and the prognostic implications of this atypical presentation.

We reviewed previous literature reports and found that the vast majority of documented cases of isolated thrombocytosis involved adult patients, with only three pediatric cases reported (9–11) (Table 3). One case involved an 11-year-old girl who presented with headaches and vomiting due to thrombocytosis. Examination revealed a platelet count exceeding 3,000 × 10^9^/L. Bone marrow cytogenetic analysis confirmed a diagnosis of CML. She achieved remission following sequential treatment with platelet exchange, hydroxyurea, anagrelide, and imatinib (9). Another pediatric case involved a 10-year-old boy with a peak platelet count of 8,688 × 10^9^/L. initially diagnosed with thrombocytosis and treated with hydroxyurea and platelet apheresis. Subsequent fusion gene testing confirmed BCR::ABL1 positivity, leading to a definitive CML diagnosis. Immediate initiation of imatinib targeted therapy ultimately achieved complete remission (10). A third patient was a 9-year-old boy who initially presented with cough and dizziness. His platelet count peaked at 3,064 × 10^9^/L. After BCR::ABL1 fusion gene testing confirmed CML, treatment with dasatinib and aspirin achieved complete remission (11). At the time of diagnosis, these patients were already experiencing symptoms such as dizziness, headache, or vomiting, which may represent early manifestations of an increased thrombotic risk due to markedly elevated platelet counts; consequently, two of them underwent plateletpheresis. In contrast to these previously reported pediatric cases, the symptoms in our patient were more insidious during the early stages of the disease. The anemia observed early in our case was attributable to prolonged menstrual cycles rather than being a direct manifestation of CML, and it resolved promptly following effective treatment. In other words, our case lacked clinical symptoms or physical signs directly associated with CML; only laboratory findings indicated thrombocytosis, which significantly complicated the diagnostic process. At the same time, the patient's chromosomal analysis revealed a normal karyotype, and the absence of typical Philadelphia chromosome-positive features greatly complicated the diagnosis. These findings emphasize that, in the absence of any typical symptoms or signs of chronic myeloid leukemia (CML), isolated thrombocytosis may be the sole precursor to the disease; routine karyotyping and morphological examination may still fail to detect it, underscoring the necessity and priority of BCR::ABL1 fusion gene testing. Clinicians must remain highly vigilant in this regard. Regarding treatment, both the previously reported cases and the case described herein achieved effective remission of the underlying disease following TKI therapy. In conjunction with other reported adult cases, CML presenting with isolated thrombocytosis generally carries a favorable prognosis. Remission is typically achievable with TKI therapy, although vigilance is warranted for complications arising from severe thrombocytosis in the early stages. For patients presenting with such complications at initial diagnosis, symptomatic measures may be administered to alleviate symptoms. However, prompt diagnosis and targeted therapy addressing the underlying etiology remain paramount.

**Table 3:** Literature review on CML characterized by isolated thrombocytosis.

**References**	**Age**	**Sex**	**Highest platelet count (×10^9^/L)**	**WBC at diagnosis (×10^9^/L)**	**Other accompanying symptoms**	**Splenomegaly**	**BCR::ABL1 transcript type**	**Philadelphia Chromosome**	**Treatment**	**Outcomes**	**Duration of follow-up/response**
Jia et al. (18)	66	Female	3,798	NA	Myocardial infarction	No	NA	Yes	Imatinib, Interferon-α	CCyR	8 months
Zhang et al. (19)	41	Female	1,742	12.12	NA	No	b3a3	Yes	Imatinib, Aspirin	MMR	3 months
Gao et al. (20)	24	Female	2,300	4.3	NA	No	NA	Yes	Imatinib	MMR	3 months
Zhang et al. (21)	54	Female	1,186	11.2	NA	No	NA	Yes	Imatinib	MMR	NA
Aktekin et al. (9)	11	Female	>3,000	13.5	Headache and vomiting	No	NA	Yes	Thrombopheresis, Low Molecular Weight Heparin, Hydroxyuream, Anagrelide, Imatinib	MMR	8 months
Findakly et al. (22)	21	Female	1,148	7.2	Vagal syncope	No	NA	Yes	Aspirin, Dasatinib	MMR	32 months
Boklan et al. (10)	10	Male	8,688	12.0	Ecchymosis	No	NA	Yes	Hydroxyuream, Thrombopheresis, Imatinib	MMR	3 months
Byun et al. (23)	21	Female	3,777	10.0	Ovarian cyst rupture	No	b3a2	Yes	Hydroxyuream, Imatinib	MMR	3 months
Ebrahem et al. (24)	39	Female	2,500	13.8	Syncope, convulsions, myocardial infarction	No	NA	NA	Hydroxyuream, Aspirin, Dasatinib	Under follow-up	NA
Breccia et al. (25)	43	Male	2,200	2.8	NA	No	e6a2	Yes	Imatinib	CHR	3 weeks
Huho et al. (11)	9	Male	3,064	7.93	Headache, cough	No	NA	Yes	Aspirin, Dasatinib	MMR	9 months

CHR, complete hematological response; CCyR, complete cytogenetic response; MMR, major molecular response.

**Figure 1:**
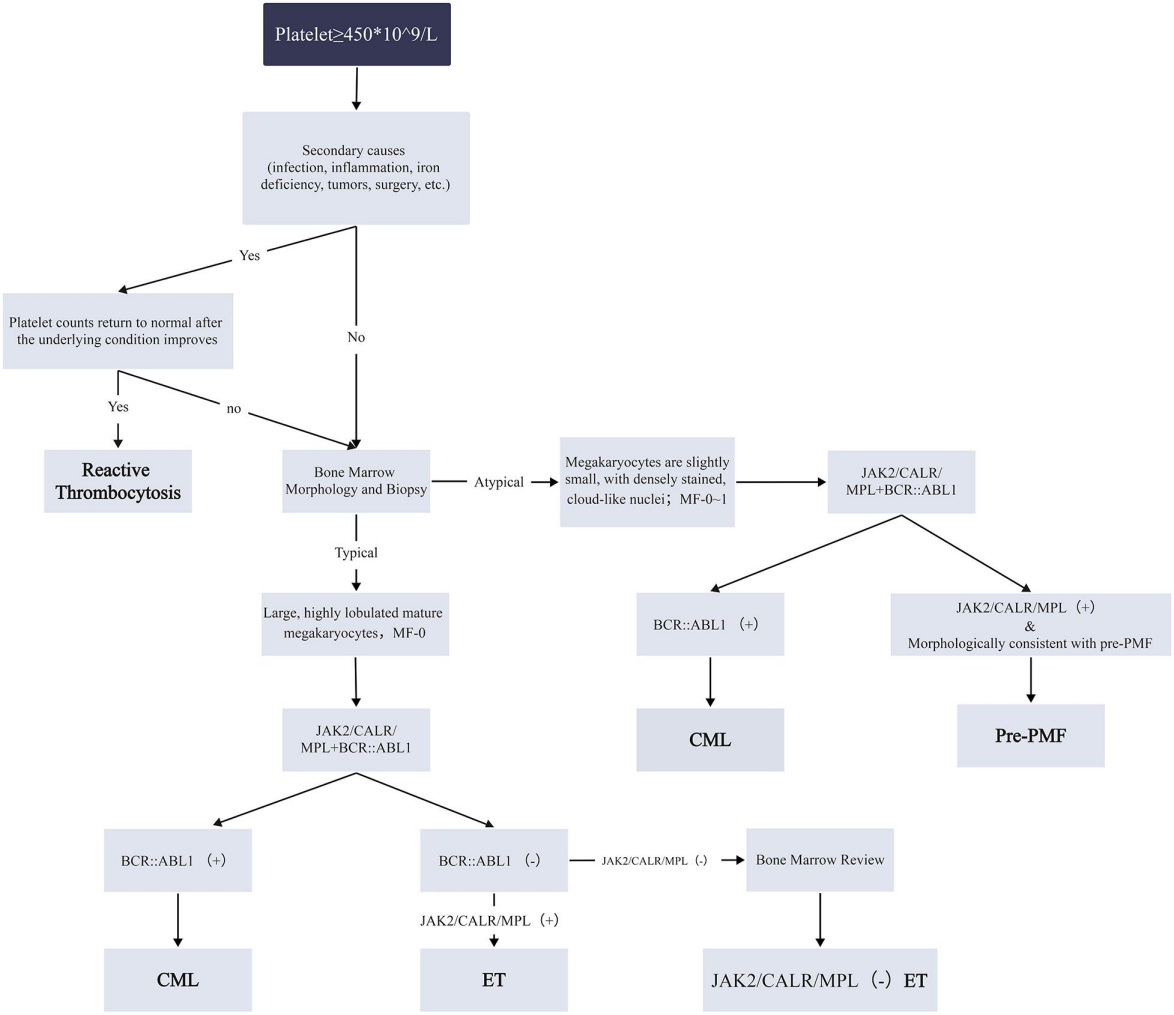
Differential diagnosis of isolated thrombocytosis in children. ET, essential thrombocythemia; CML, Chronic myeloid leukemia; pre-PMF, prefibrotic primary myelofibrosis.

For the diagnosis of this type of CML, the most critical distinction is from essential thrombocythemia (ET) (Figure 1). ET is a clonal myeloproliferative neoplasm characterized by excessive platelet production. Over 80% of ET cases habor genetic mutations that activate the JAK-STAT pathway, including JAK2, CALR, and MPL (12). Clinically, preliminary differentiation between ET and CML can be achieved through bone marrow morphology examination. ET bone marrow typically shows abundant megakaryocytes, generally distributed in loose clusters, with mostly normal proportions of granulocytes and erythrocytes. In contrast, CML megakaryocytes are smaller in morphology compared to those in ET (13). However, bone marrow morphology alone is often insufficient for definitive differentiation. Thus, prompt genetic testing is essential for pediatric patients with unexplained thrombocytosis. It is important to note that a minority of ET cases may lack mutations in JAK2, CALR, or MPL. Furthermore, extremely rare instances of ET may transform into CML, clinically presenting with concurrent ET-associated genetic alterations and the BCR::ABL1 fusion gene (14, 15). Regarding treatment, hydroxyurea is the first-line therapy for ET, and the majority of ET patients respond well to it (16, 17). In our case, due to atypical clinical symptoms and limitations in outpatient testing at the initial visit, BCR::ABL1 fusion gene testing was not performed in a timely manner. The possibility of CML was only considered after empirical treatment with hydroxyurea and aspirin proved ineffective. Therefore, we strongly recommend that pediatric patients with unexplained thrombocytosis undergo concurrent testing for the BCR::ABL1 fusion gene, chromosomal karyotyping, and ET-related genes (e.g., JAK2, CALR, MPL) to minimize the risk of missing a diagnosis of CML with atypical clinical presentation. If testing resources are limited and ET cannot be ruled out, empirical treatment with hydroxyurea may be initiated; however, platelet counts should be closely monitored during treatment. If the response is suboptimal, chromosomal karyotyping and fusion gene testing should be promptly performed.

However, this study has certain limitations. Since the patient is still undergoing treatment and follow-up, and is currently responding well to therapy, it is not yet possible to assess the long-term prognosis. Given the rarity of such cases, it is currently difficult to estimate the true incidence of this CML subtype in children. Further real-world studies are needed to characterize the epidemiological features of this type of CML.

## Conclusion

Isolated thrombocytosis can be the sole manifestation of CML, although this is extremely rare in clinical practice. The clinical presentation of this CML subtype is insidious, rendering diagnosis challenging. At initial evaluation, careful distinction from ET is essential to avoid delays in treatment due to misdiagnosis or missed diagnosis. Therefore, we propose that BCR::ABL1 fusion gene testing should be considered an essential component of the initial diagnostic workup for any child with persistent, unexplained thrombocytosis, particularly before initiating long-term ET-directed therapy. Current evidence indicates that patients with this CML variant have a favorable prognosis; once a definitive diagnosis is established, they should be promptly started on TKIs therapy, targeting the BCR::ABL1 oncoprotein, to achieve early and deep remission.

## Data Availability

The original contributions presented in the study are included in the article, further inquiries can be directed to the corresponding author.
